# Use of the Medtronic Hugo Robot-Assisted Surgery (RAS) System in Urogynecology: A Safety and Feasibility Study of the First 100 Procedures

**DOI:** 10.7759/cureus.72213

**Published:** 2024-10-23

**Authors:** Meera Ragavan, Deerush Kannan, Amrithavarshini Ragavan, Madhav Tiwari, Narasimhan Ragavan

**Affiliations:** 1 Urogynecology, Apollo Hospitals, Chennai, IND; 2 Urology, Apollo Hospitals, Chennai, IND

**Keywords:** hugo ras, hugo robot, pelvis surgery, robotic-assisted surgery, urogynecology

## Abstract

Introduction: This study evaluates the safety and feasibility of the Medtronic Hugo robot-assisted surgery (RAS) system in gynecological and urogynecological procedures. Through a retrospective analysis of prospectively collected data from the first 100 surgeries performed at Apollo Hospitals Chennai, we aim to provide insights into the system’s effectiveness, including outcomes, complications, and overall surgical efficiency in major gynecological and urogynecological operations.

Methods: A single experienced robotic surgeon conducted the surgeries, including hysterectomies (n=66). Other common procedures performed were for pelvic organ prolapse (n=12), vesicovaginal fistula (VVF) (n=8), and ovarian cystectomies (n=8). Data on patient demographics, peri and postoperative outcomes, complications, and 30-day readmission rates were collected and analyzed.

Results: All surgeries were completed without open conversions. One patient experienced a sigmoid colon injury intraoperatively due to severe adhesions from endometriosis; the injury was repaired intraoperatively, and the patient had an uneventful recovery. No patients experienced Clavien-Dindo grade 2 postoperative complications, and there were no readmissions within 30 days. The median docking time was 10.4 ± 2.2 minutes, console time was 60.5 ± 19.7 minutes, and hospital stays ranged from 1 to 4 days, all within acceptable ranges.

Conclusions: The Medtronic Hugo RAS system demonstrates safety and feasibility in major urogynecology procedures, suggesting its potential as a valuable addition to robotic surgical platforms.

## Introduction

Robot-assisted surgery (RAS) has revolutionized minimally invasive procedures, offering enhanced precision, reduced recovery times, and improved surgical outcomes [1]. In urogynecology, the adoption of robotic systems has been particularly transformative, addressing complex conditions such as pelvic organ prolapse and vesicovaginal fistulas with greater efficacy and safety [2]. The Medtronic Hugo RAS system, a recent innovation in this field, integrates advanced robotic technology with surgeon-friendly features, promising to further enhance surgical performance and patient outcomes [3].

The Hugo RAS system is designed to overcome the limitations of earlier robotic platforms, featuring modular components, improved ergonomics, and enhanced visualization capabilities [3]. These advancements are crucial for delicate gynecological and urogynecological surgeries, where precision and control are paramount [4,5]. Despite the growing enthusiasm for robotic systems, comprehensive studies on the safety and feasibility of the Hugo RAS system in urogynecology remain limited.

This study aims to provide a comprehensive retrospective analysis of gynecological and urogynecological procedures performed with the Hugo RAS system at a tertiary care referral center in India. By evaluating perioperative metrics, complication rates, and patient outcomes, we aim to establish the system’s effectiveness and reliability in clinical practice. Our findings will contribute to the ongoing assessment of robotic technologies in urogynecology, guiding future surgical practices and innovations.

## Materials and methods

Study design and setting

This retrospective analysis of the prospectively collected database was conducted at the Department of Urogynecology, Apollo Hospitals Chennai, focusing on gynecological and urogynecological surgeries performed using the Medtronic Hugo RAS system between September 2021 and December 2023. A robust data collection has been conducted at the institute for the performance and safety of the system since its establishment. 

Patient selection

A total of 100 patients undergoing various gynecological and urogynecological procedures were included in the study. Patients were selected based on the indication for surgery and suitability for robotic-assisted procedures, as determined by the attending surgeon. All patients provided informed consent prior to surgery. These 100 patients were part of the initial 100 surgeries performed with this robotic system. 

Surgical procedures

An experienced robotic surgeon conducted all surgeries, which included hysterectomies (n=66), surgeries for pelvic organ prolapse (n=12), vesicovaginal fistula (VVF) repairs (n=8), ovarian cystectomies (n=8), and other urogynecological procedures (n=6). The Medtronic Hugo™ RAS system (Medtronic, Minneapolis USA) was utilized for all surgeries, with specific attention to docking time, console time, blood loss, perioperative complication, and hospital stay.

Data collection

Data on patient demographics (age, body mass index, comorbidities), perioperative outcomes (docking time, console time, blood loss, duration of hospital stay), postoperative outcomes, complications, and 30-day readmission rates were collected from medical records. The additional data collected included intraoperative complications such as bleeding, organ injury, and other events, along with details of conversions to open or laparoscopic surgery and any technical issues with instruments leading to adverse or near-miss events. Postoperative data covered recovery metrics like time to first ambulation, bowel movement, and resumption of a normal diet. Functional outcomes were assessed based on improvements in urogynecological symptoms during follow-up visits, while complications beyond the initial 30 days, extending up to three months post-surgery, were monitored. Additionally, recurrence rates of the original condition that necessitated surgery were tracked.

Statistical analysis

Descriptive statistics were used to summarize patient demographics and perioperative outcomes. Continuous variables were presented as means and standard deviations or medians and interquartile ranges, as appropriate. Categorical variables were summarized as frequencies and percentages. The incidence of complications, conversions to open surgery, and readmissions within 30 days post-surgery were also reported.

Ethical considerations

The study was approved by Institutional Ethics Committee - Biomedical Research (AMH-C-S-034/05-24), Apollo Hospitals, Chennai. All procedures performed in this study involving human participants were in accordance with the ethical standards of the institutional and national research committee and with the 1964 Helsinki Declaration and its later amendments or comparable ethical standards.

## Results

The various demographic and surgical parameters of the study population are tabulated in Table 1 and Table 2.

**Table 1:** Descriptive factors Continuous variables presented as means and standard deviations

Parameters	Values
Age (in years)	
Mean ± SD	50.7 ± 15.2
Range	23 – 81
20 – 40 years	26 (26%)
41 – 60 years	45 (45%)
61 – 80 years	28 (28%)
> 80 years	1 (1%)
Body mass index	
Mean ± SD	22.9 ± 1.4
Range	19 – 28
Normal	97 (97%)
Overweight	3 (3%)
Hospital stay	
Mean ± SD	1.4 ± 0.9
Range	1 – 4 days
1 day	81 (81%)
2 days	6 (6%)
> 2 days	13 (13%)

**Table 2:** Surgical factors Continuous variables presented as means and standard deviations

Surgery	n =100 (%)
Hysterectomies	66 (66)
Ovarian cystectomy	8 (8)
Vesicovaginal fistula repair	8 (8)
Sacrocolpopexy	4 (4)
Ileopectopexy	4 (4)
Colpopectopexy	4 (4)
Others	6 (6)
Dock time in mins	
Mean ± SD	10.4 ± 2.2
Range	8 – 20
<10 Mins	85 (85)
>10 Mins	15 (15)
Console time in mins	
Mean ± SD	60.5 ± 19.7
Range	30 – 120
<60 Mins	69 (69)
>60 Mins	31 (31)

The majority (45%) of the study population (n=45) belonged to the perimenopausal age group of 40 to 50 years. Three out of 100 patients were overweight with a body mass index (BMI) of more than 25. Eighty-one out of the 100 patients were discharged on the first postoperative day, with none of the patients requiring readmission for any surgical cause within 30 days of surgery. There were no intraoperative complications, open conversions, or requirements for blood transfusion (no Clavien-Dindo grade II complications).

**Figure 1:**
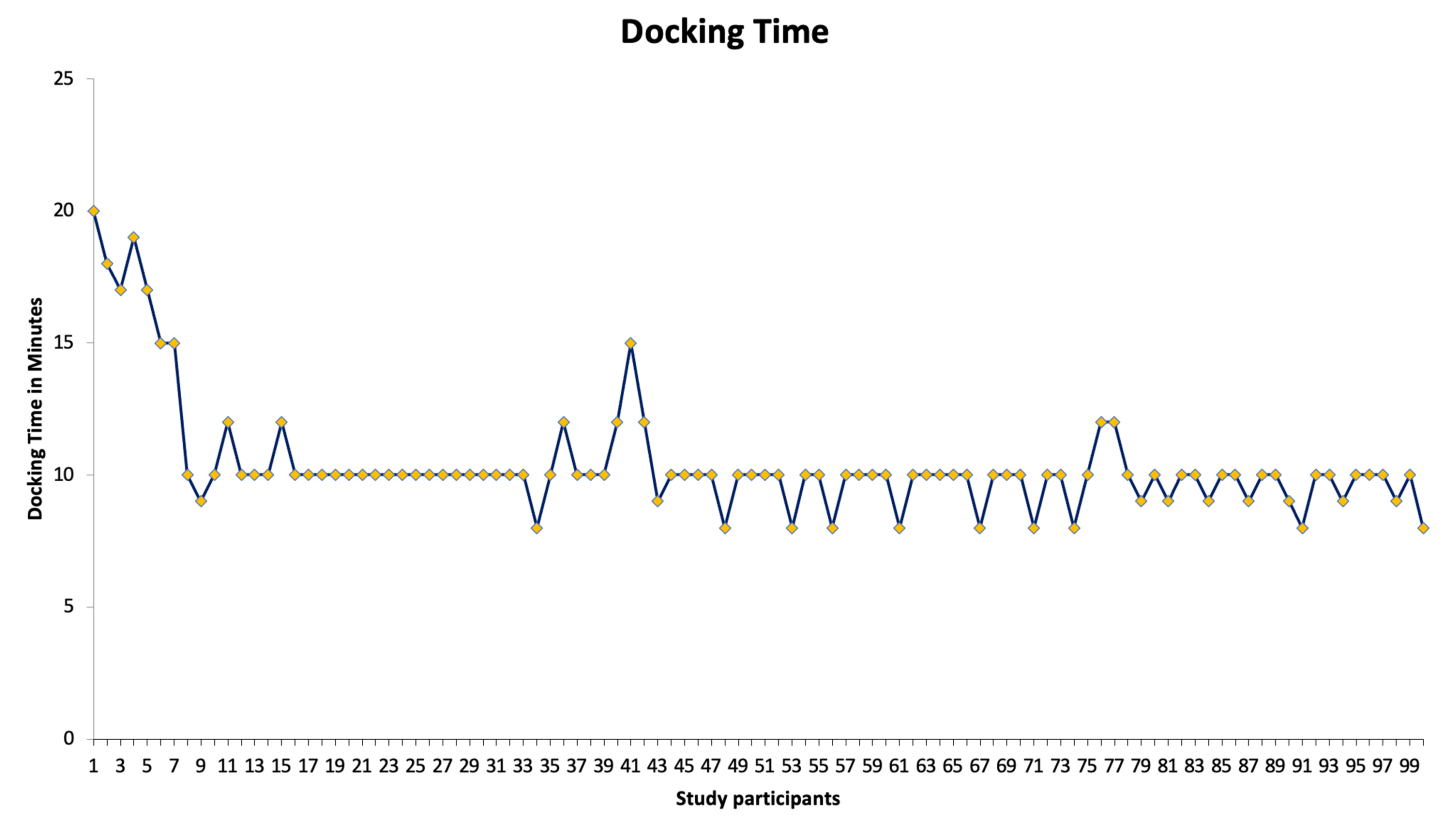
Line chart depicting docking times across 100 procedures

**Figure 2:**
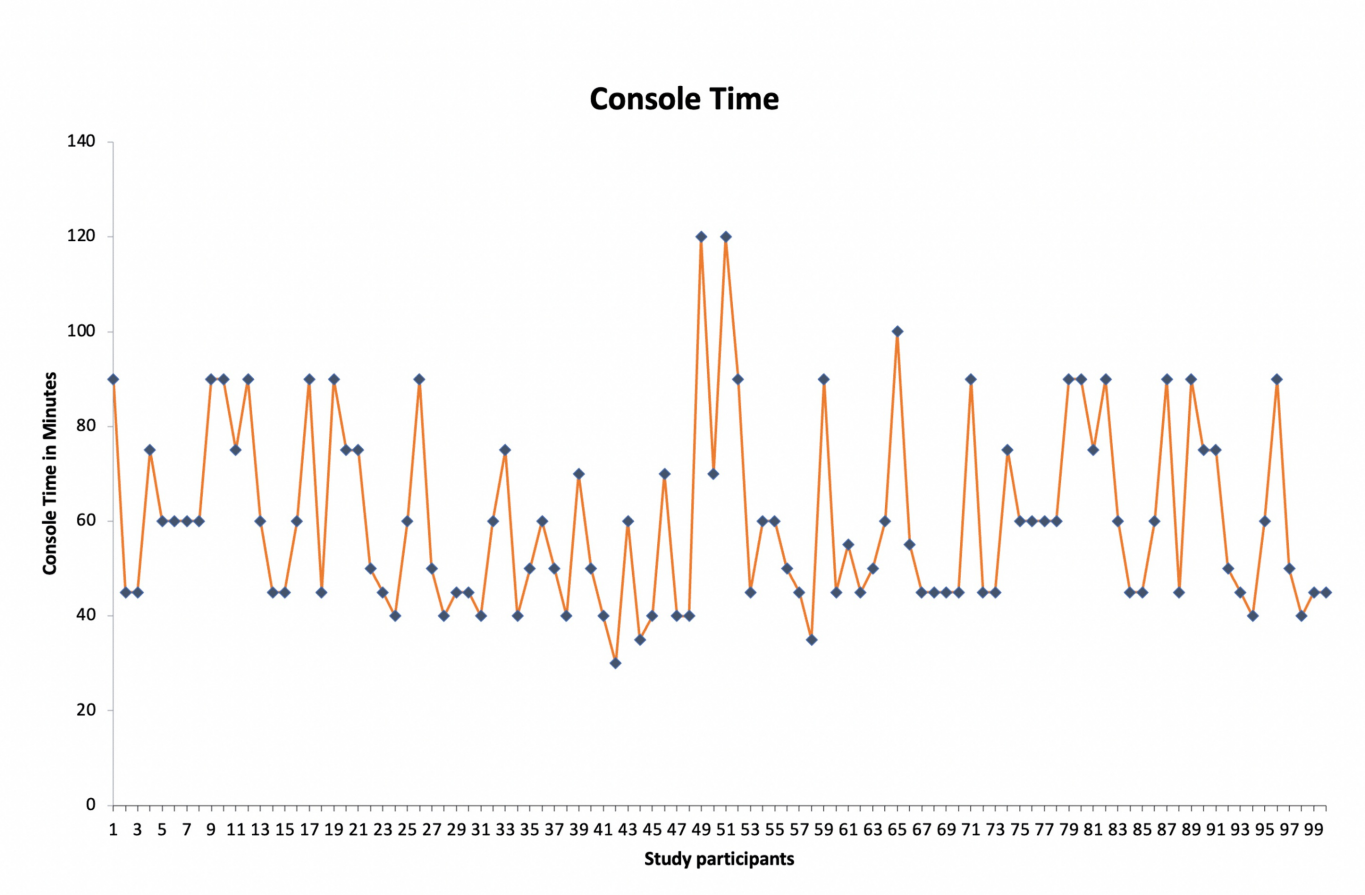
Line chart depicting console time across various procedures

Hysterectomy (n=66) was the most commonly performed procedure (66%), and the docking times reached a nadir of around 10 minutes at case 10. Out of the 66 hysterectomies, 11 were performed for endometrial carcinoma and this set of patients (n=11) required nodal dissection that added to the intraoperative times. There was an increase in docking time again at cases 39 to 43 when a new team member was scrubbed for the procedure (Figure 1). The console time varied depending on the type of surgery performed, as displayed in the line chart (Figure 2).

## Discussion

The use of RAS in urogynecology has been transformative, enhancing precision and patient outcomes [6]. Our experience with the Medtronic Hugo RAS system underscores both the initial challenges and benefits of integrating new robotic technology into surgical practice [7]. Initially, our team, including the bedside assistant, and other team members, faced a learning curve. However, after approximately 10 cases, we adapted significantly. Modifications in practice, such as optimizing port placement and the assistant port, allowed us to perform procedures more ergonomically with fewer instrument clashes. The dock time decreased and reached a nadir after 10 cases which can be appreciated on the graph. The console time did not require a learning curve probably because the surgeon was already trained with the other robotic platforms and underwent multiple settings of practice on the console as analogous to the learning curve studied by Soomro et al. [6]. 

Interestingly, despite the initial learning phase, we did not observe an increase in complications. This finding aligns with existing literature, which often notes that complications typically do not rise during the learning period of robotic systems [8]. The only intraoperative complication noticed was a sigmoid colon injury during a hysterectomy for endometriosis. The injury occurred due to severe adhesions secondary to endometriosis, not due to a system error. However, the patient made an uneventful recovery after prompt recognition and repair of the injury intraoperatively. The primary challenge we encountered was an extended operative time, especially with docking. Over time, as our familiarity with the Hugo system improved, docking times decreased, underscoring the importance of consistent training and having a dedicated team. Studies have shown that a well-trained team can significantly reduce operative times and improve efficiency [9].

We could figure out the angles at which the arms of the Hugo RAS system have to be docked for effective movement of the arms without clashes. At the same time, the placement of the assistant ports (one 12 mm and one 5 mm port) was learned out of experience after the initial 15 cases that could help the assistant to work with ease from the bedside.

Our parameters, including intraoperative and postoperative outcomes, were consistent with those reported for other robotic platforms. Notably, our practice of Enhanced Recovery After Surgery protocols ensured that postoperative outcomes with the Hugo system were comparable to those achieved with the da Vinci system [10]. This suggests that the Hugo RAS system is a viable alternative to established robotic platforms in terms of intraoperative safety, efficacy, and patient recovery and outcomes with a note that the dock times could be higher for the initial 10 cases based on our experience. 

The surgeon's prior extensive experience with the da Vinci system facilitated a smoother transition to the Hugo system. The foundational skills and robotic surgery principles gained from da Vinci were transferable, allowing for quicker adaptation and proficiency with the new system. This highlights the benefit of cross-platform robotic surgery training [11].

However, this study has limitations. We did not perform a comparison of the console time of the surgeon as performed on other robotic platforms. This study represents the experience of a single, highly experienced surgeon at a tertiary center, which may not reflect the broader patient population or the experiences of new surgeons adopting the system. Therefore, results may vary in less experienced hands or different clinical settings.

## Conclusions

The Medtronic Hugo RAS system proves to be a safe and effective tool for gynecological and urogynecological procedures. Despite an initial learning curve, particularly with docking times, no increase in complications was observed. Consistent training and a dedicated team are critical in optimizing the use of this robotic platform. Future studies should explore multisurgeon and multicenter experiences to validate these findings further.
